# Characterization of ferroptosis in kidney tubular cell death under diabetic conditions

**DOI:** 10.1038/s41419-021-03452-x

**Published:** 2021-02-08

**Authors:** Seonghun Kim, Shin-Wook Kang, Jeongho Joo, Seung Hyeok Han, Huiyoon Shin, Bo Young Nam, Jimin Park, Tae-Hyun Yoo, Gyuri Kim, Pureunchowon Lee, Jung Tak Park

**Affiliations:** 1 Department of Oral Pathology, Oral Cancer Research Institute, College of Dentistry, Yonsei University, Seoul, South Korea; 2 Institute of Kidney Disease Research, College of Medicine, Yonsei University, Seoul, South Korea; 3 Department of Internal Medicine, College of Medicine, Yonsei University, Seoul, South Korea; 4 Genome & Health Big Data Laboratory, Seoul National University, Seoul, South Korea; 5 Severance Biomedical Science Institute, College of Medicine, Yonsei University, Seoul, South Korea

**Keywords:** Cell death, Kidney diseases

## Abstract

Kidney tubular cell death induced by transforming growth factor-β1 (TGF-β1) is known to contribute to diabetic nephropathy, a major complication of diabetes. Caspase-3-dependent apoptosis and caspase-1-dependent pyroptosis are also involved in tubular cell death under diabetic conditions. Recently, ferroptosis, an atypical form of iron-dependent cell death, was reported to cause kidney disease, including acute kidney injury. Ferroptosis is primed by lipid peroxide accumulation through the cystine/glutamate antiporter system X_c_^−^ (xCT) and glutathione peroxidase 4 (GPX4)-dependent mechanisms. The aim of this study was to evaluate the role of ferroptosis in diabetes-induced tubular injury. TGF-β1-stimulated proximal tubular epithelial cells and diabetic mice models were used for in vitro and in vivo experiments, respectively. xCT and GPX4 expression, cell viability, glutathione concentration, and lipid peroxidation were quantified to indicate ferroptosis. The effect of ferroptosis inhibition was also assessed. In kidney biopsy samples from diabetic patients, xCT and GPX4 mRNA expression was decreased compared to nondiabetic samples. In TGF-β1-stimulated tubular cells, intracellular glutathione concentration was reduced and lipid peroxidation was enhanced, both of which are related to ferroptosis-related cell death. Ferrostatin-1 (Fer-1), a ferroptosis inhibitor, alleviated TGF-β1-induced ferroptosis. In diabetic mice, kidney mRNA and protein expressions of xCT and GPX4 were reduced compared to control. Kidney glutathione concentration was decreased, while lipid peroxidation was increased in these mice, and these changes were alleviated by Fer-1 treatment. Ferroptosis is involved in kidney tubular cell death under diabetic conditions. Ferroptosis inhibition could be a therapeutic option for diabetic nephropathy.

## Introduction

Diabetic nephropathy, a severe microvascular complication of diabetes, is characterized by proteinuria and a progressive decline in kidney function, leading to end-stage kidney disease^1,2^. The pathological features of diabetic nephropathy include extracellular matrix (ECM) accumulation within the glomeruli and tubulointerstitium and glomerular and tubular cell death, all of which contribute to the development of kidney fibrosis and tubular atrophy^3–5^. Accumulating evidence indicates that high glucose, hemodynamic changes, and local growth factors are all involved in the pathogenesis of diabetic nephropathy^6–10^. In particular, transforming growth factor-β (TGF-β) is regarded as one of the main mediators of the deleterious effects of the diabetic milieu, and is known to mediate kidney fibrosis and tubular cell death under diabetic conditions^11–13^. Meanwhile, a number of previous studies have shown that caspase-3-dependent apoptosis and caspase-1-dependent pyroptosis are also involved in tubular cell death in diabetic nephropathy^14,15^.

A new atypical form of cell death, ferroptosis, was recently identified. Ferroptosis, iron-dependent cell death, is different from apoptosis, pyroptosis, and receptor-interacting protein kinase-dependent necroptosis^16^. The key mediators of ferroptosis are cystine/glutamate antiporter system X_c_^−^ (xCT) and glutathione peroxidase 4 (GPX4), an antioxidant enzyme. Low xCT and GPX4 levels reduce intracellular cystine concentrations, which in turn diminish glutathione synthesis, and lipid peroxide degradation, respectively. Both of these changes result in the accumulation of intracellular lipid peroxide, which leads to ferroptosis^17–19^.

Recent studies revealed that ferroptosis is involved in tubular cell death during acute kidney injury (AKI)^18,20^. Ferrostatin-1 (Fer-1), a small-molecule compound that inhibits lipid oxidation and subsequently suppresses ferroptosis, has been shown to abrogate tubular injury in experimental AKI models^21,22^. In addition, tubular cell death and interstitial edema within the kidney and proteinuria were significantly increased in GPX4-null mice, suggesting a reno-protective role for GPX4 in tubular cells^23^. In contrast to AKI, however, the impact of ferroptosis on the development of diabetic nephropathy has not been elucidated.

Therefore, the aim of the current study was to investigate the role of ferroptosis in tubular cell death under diabetic conditions. To test our hypothesis, we used TGF-β-stimulated cultured kidney tubular cells and diabetic mice models for in vitro and in vivo experiments, respectively. Furthermore, we also examined the effect of ferroptosis inhibition on diabetes-induced tubular cell injury.

## Materials and methods

### Human kidney sample collection

Kidney biopsy samples were obtained from 44 adult patients who underwent biopsy procedures due to medical indications at Yonsei Medical Center, Seoul, Korea. Renal biopsy was performed in the standard manner^24^. Microdissection of the kidney biopsy samples were performed, as previously described^25^. Kidney tubules, with glomeruli removal through microdissection, was stored in RNAiso reagent at −70 °C (Takara Bio Inc., Otsu, Shiga, Japan). Samples from type 2 diabetic patients whose kidney biopsy pathological diagnosis revealed diabetic nephropathy were allocated as diabetes mellitus (DM). Non-DM samples were from those without DM and whose pathological findings did not show specific abnormalities or only minimal glomerular change. Kidney tissues were collected with the approval from the Institutional Review Board for human research at Yonsei University College of Medicine and informed consent was obtained from all patients (4-2006-0154).

### Cell culture and treatment of NRK-52E cells

NRK-52E cells, immortalized rat proximal tubular epithelial cells, were maintained in DMEM supplemented with 5% fetal bovine serum (FBS), 100 U/ml penicillin, 100 mg/ml streptomycin, and 26 mM NaHCO_3_, and were grown at 37 °C in humidified 5% CO_2_ in air. The medium was changed to DMEM containing 5.6 mM glucose 48 h after seeding. Subconfluent NRK-52E cells were FBS-restricted for 24 h, then the medium was replaced with 1% FBS DMEM for the control group and the same medium plus TGF-β1 (10 ng/ml; R&D Systems, Minneapolis, MN, USA) for the TGF-β1 group. Both groups were treated with Fer-1 (1–100 μM; Selleckchem, Houston, TX, USA). NRK-52E cells were harvested 12 h after treatment. Cells were stimulated with Erastin (Selleckchem) for positive control ferroptosis induction.

### Animal experiments

All animal protocols were approved by the Committee for the Care and Use of Laboratory Animals at Yonsei University College of Medicine in Seoul, Korea. All experiments were conducted in accordance with the Principles of Laboratory Animal Care (NIH Publication no. 85-23, revised 1985). Forty C57BL/6 mice weighing 20–25 g were injected with either diluent [*n* = 20, control (Con)] or 50 mg/kg streptozotocin (STZ; Sigma-Aldrich, St. Louis, MO, USA; *n* = 20, STZ) intraperitoneally for five consecutive days. Tail vein blood glucose levels were measured to confirm DM (fasting blood glucose > 300 mg/dl). Ten mice from each group were treated with Fer-1 (5 mg/kg/day) via Alzet osmotic pumps (DURECT Corporation, Cupertino, CA, USA). Mice were housed in a temperature-controlled room and given free access to water and standard laboratory chow during the 12-week study period. After 12 weeks, mice were anesthetized with Zoletil (10 mg/kg; Virbac, Carros, France), and kidneys were extracted after sacrifice. Body weight, kidney weight, blood glucose, blood urea nitrogen (BUN), serum creatinine, 24-h urinary albumin, and creatinine concentrations were determined at the time of sacrifice. Blood glucose was measured with a glucometer, and 24-h urinary albumin was assessed via enzyme-linked immunosorbent assay (ELISA; Nephrat II, Exocell Inc., Philadelphia, PA, USA). BUN, serum creatinine, and 24-h urinary creatinine were analyzed using a Cobas 8000 C702 (Roche, Mannheim, Baden Württemberg, Germany). Type 2 DM *db/db* mice and genetic control non-DM *db/m* mice (6 weeks old, three per group) were obtained from Jackson Laboratories (Bar Harbor, ME, USA) and were sacrificed after 12 weeks.

### Quantitative real-time polymerase chain reaction

Total RNA purification, reverse transcription, and real-time polymerase chain reaction (PCR) of renal biopsy samples, NRK-52E cells, and whole kidney samples were performed, as previously described^26^. A total reaction volume of 20 μl per well was used, including 25 ng RNA and 10 μl SYBR Green PCR Master Mix (Applied Biosystems). PCR conditions consisted of initial heating for 9 min at 95 °C, followed by 40 cycles of denaturation for 30 s at 94.5 °C, annealing for 30 s at 60 °C, and extension for 1 min at 72 °C, and a final extension for 7 min at 72 °C. PCR was performed using an ABI PRISM 7700 Sequence Detection System (Applied Biosystems, Foster City, CA, USA). Primer sequences used for real-time PCR were: *Slc7a11* (human), forward 5′-CCCAGATATGCATCGTCCTT-3′ reverse 5′-CCTGGGTTTCTTGTCCCATA-3′; *Slc7a11* (rat), forward 5′-TGAATGCCTTGTCTGCTTTG-3′ reverse 5′-GAATTGCAGGGAACTGTGGT-3′; *Slc7a11* (mouse), forward 5′-GATGCTGTGCTTGGTCTTGA-3′ reverse 5′-GCCTACCATGAGCAGCTTTC-3′; *Gpx4* (human), forward 5′-AGATCCACGAATGTCCCAAG-3′ reverse 5′-CCTCCTCCTTAAACGCACAC-3′; *Gpx4* (rat, mouse), forward 5′-CCGGCTACAATGTCAGGTTT-3′ reverse 5′-ACGCAGCCGTTCTTATCAAT-3′; *Fth1* (rat), forward 5′-ATGATGTGGCCCTGAAGAAC-3′ reverse 5′-TCATCACGGTCAGGTTTCTG-3′; *Fth1* (mouse), forward 5′-GCCGAGAAACTGATGAAGCTGC-3′ reverse 5′-GCACACTCCATTGCATTCAGCC-3′; 18 s (human), forward 5′-ACCGCGGTTCTATTTTGTT-3′ reverse 5′-CGGTCCAAGAATTTCACCTC-3′; 18 s (rat), forward 5′-CAAGTAGGAGAGGAGCGAGC-3′ reverse 5′-CATGTCTAAGTACGCACGGC-3′; 18 s (mouse), forward 5′-CGCTTCCTTACCTGGTTGAT-3′ reverse 5′-GGCCGTGCGTACTTAGACAT-3′.

cDNA content of each specimen was determined using a comparative *C*_T_ method with 2^−△△CT^. Results are shown as relative expression normalized to 18 s rRNA expression with arbitrary units.

### Western blot analysis

Western blotting was performed as previously described^26^. Primary antibodies were polyclonal antibodies to xCT (NB300-318, Novus Biologicals, Littleton, CO, USA), GPX4 (NBP2-75511, Novus Biologicals), ferritin heavy chain 1 (FTH1, #3998, Cell Signaling Technology, Danvers, MA, USA), p53 (#9282 S, Cell Signaling Technology), NRF2 (NBP1-32822, Novus Biologicals), β-actin (A5441, Sigma-Aldrich), or Lamin B1 (ab133741, Abcam). Membranes were washed three times for 10 min in TBS with 0.1% Tween-20 then incubated in buffer A containing a 1:1000 dilution of horseradish peroxidase-conjugated goat-anti rabbit, or anti-mouse IgG (GenDEPOT, Barker, TX). ImageJ software (NIH, Bethesda, MD, USA) was used to measure band intensities, and changes in treated groups relative to control cells or tissues were used for analysis.

### Cell viability assay

Cell viability was assessed using a 3-(4,5-dimethylthiazol-2-yl)-2,5-diphenyltetrazolium bromide (MTT) assay kit (Thermo Fisher Scientific, Waltham, MA, USA). NRK-52E cells were seeded onto 96-well plates (3 × 10^3^ per well) and FBS-restricted for 24 h. Subsequently, cells were treated with various concentrations of TGF-β1 or Fer-1. Cell viability was determined at different time points after treatment (usually 12 h, unless stated otherwise). Fresh medium containing 0.5 mg/ml MTT solution was added to each well and incubated for 4 h at 37 °C. The supernatant was aspirated, and 100 μl DMSO (Sigma-Aldrich) was added. Absorbance was measured at 570 nm using a VersaMax ELISA Microplate Reader (Molecular Devices, Silicon Valley, CA, USA).

### Iron assay

Intracellular and tissue levels of total iron were assessed using an iron assay kit (Sigma-Aldrich). Cultured cells (1 × 10^6^ cells) or mouse kidney tissue samples (10 mg) were harvested and homogenized in iron assay buffer. All samples were centrifuged at 16,000 × *g* for 10 min. A total of 10 μl of supernatant from each sample was added to 90 μl of the iron assay buffer. Subsequently, 5 μl of iron reducer was added to each supernatant. The mixture was incubated for 30 min, and total iron levels were determined using iron probe at a wavelength of 593 nm.

### Glutathione assay

Glutathione concentration was assessed using a Glutathione Assay Kit (Sigma-Aldrich). Cultured cells and mouse kidneys were harvested and lysed by repeated freeze–thaw cycles in 5% 5-sulfosalicylic acid solution and centrifuged at 10,000 r.p.m. for 10 min. A total of 10 μl of supernatant from each sample was added to 150 μl of the working mixture (glutathione reductase and DTNB solution) and incubated for 5 min at RT. NADPH solution was added to each well, and glutathione levels were determined by kinetic absorbance measurement (1 min intervals for 5 min) at a wavelength of 412 nm. The glutathione content of each specimen was calculated by comparison with the standard.

### Measurement of lipid peroxidation

Lipid peroxidation (malondialdehyde, MDA) was assessed using a lipid peroxidation assay kit (Abcam, Cambridge, United Kingdom) and an Image-iT^®^ Lipid Peroxidation Kit (Thermo Fisher Scientific) for live cells. For the MDA assay, cultured cells and whole mouse kidneys were harvested and lysed in lysis solution (MDA lysis buffer and 5% butylated hydroxytoluene) then centrifuged at 13,000 r.p.m. for 10 min at 4 °C. Supernatants were removed, and MDA levels were determined using the reaction of thiobarbituric acid at a wavelength of 532 nm. The MDA content of each specimen was calculated by comparison with the standard. For the Image-iT^®^ lipid peroxidation analysis, cells were seeded onto two-well chamber slides (2 × 10^4^ per well) and treated with TGF-β1 or Fer-1, after 24 h. The Image-iT^®^ lipid peroxidation sensor was added to each well and incubated for 30 min at 37 °C. Supernatants were removed, and samples were washed three times with PBS. Images were analyzed using an LSM700 confocal microscope (Carl Zeiss Vision, Hallbergmoos, Germany) under ×40 magnification.

### Flow cytometry with C11-BODIPY probe

In order to assess the lipid peroxidation accumulation, fluorescence-activated cell sorting (FACS) was used with C11-BODIPY probe. NRK-52E cells were seeded in six-well plates. After TGF-β1 stimulated for 12 h, cells were stained with C11-BODIPY (581/591) at 37 °C for 30 min. After washing and resuspension in FACS buffer (PBS with 5% FBS), stained cells were analyzed with LSRII using FACS Diva software (BD Bioscience, San Jose, CA, USA) and analyzed using FlowJo software, version 10.2 (TreeStar, San Carlos, CA, USA).

### Transmission electron microscopy

TGF-β1 or Erastin-stimulated NRK-52E cells with or without Fer-1 treatment were fixed for 12 h in 2% glutaraldehyde (Merck Millipore, Darmstadt, Germany)–2% paraformaldehyde (Merck Millipore) in 0.1 M phosphate buffer (pH 7.4) and washed in 0.1 M phosphate buffer, postfixed with 1% OsO4 (Polysciences) in 0.1 M phosphate buffer for 2 h and dehydrated with an ascending ethanol series. Cells were then embedded, mounted, and stained with toluidine blue for optical microscope observation. Images were captured with a transmission electron microscopy (JEOL, Tokyo, Japan) with a Megaview III CCD camera (Soft imaging system, Germany).

### Immunohistochemistry

Kidney samples were fixed in 10% neutral-buffered formalin and processed to 5 μm thick sections for immunohistochemistry. Tissue sections were deparaffinized, rehydrated in ethyl alcohol, and washed in tap water. Antigen retrieval was performed using 10 mM sodium citrate buffer for 20 min in a Black & Decker vegetable steamer. Slides were blocked with 10% donkey serum for 30 min at RT, and then washed with PBS. Primary antibodies against xCT (Novus Biologicals), GPX4 (Novus Biologicals), 4-hydroxynonenal (4-HNE; ab46545, Abcam), or MDA (ab6463, Abcam) were diluted to the appropriate concentration with 2% casein in bovine serum albumin, added to the slides, and incubated overnight at 4 °C. After washing, the secondary antibody (Dako, Carpinteria, CA, USA) was applied for 1 h at RT. Diaminobenzidine was added for 2 min, and sections were counterstained with hematoxylin. A semiquantitative score for staining intensity was obtained by examining at least five fields per section under ×20 magnification and with digital image analysis (MetaMorph version 4.6r5, Universal Imaging Corp., Downingtown, PA, USA).

### Terminal deoxynucleotidyl transferase dUTP nick-end labeling assay

Cell death was evaluated using a terminal deoxynucleotidyl transferase dUTP nick-end labeling (TUNEL) assay with a commercially available kit (Merck Millipore, Darmstadt, Germany). TUNEL-positive tubular cells were identified in formalin-fixed kidney tissue by examining at least 15 regions at ×20 magnification. Data are expressed as the number of positively stained nuclei (indicating apoptotic cells) per field.

### NE-PER nuclear and cytoplasmic extraction

Cells and kidney tissue samples were collected, cut into small pieces, washed with PBS, and then homogenized with beads in certain volume of cytoplasmic extraction reagent (CER) I in NE-PER Nuclear and Cytoplasmic Extraction Reagents kit (ThermoFisher, Waltham, MA) according to the weight of kidneys. After 15 s of vigorous vortexing and 10 min of incubation on ice, CER II was added to each kidney samples followed by incubation on ice for 1 min and centrifugation at 16,000 × *g* for 5 min. The supernatant was collected as cytoplasmic extract, and the insoluble fraction was suspended in nuclear extraction reagent. After 10 min of centrifugation at 16,000 × *g*, each supernatant was collected and used as nuclear extract.

### Statistical analysis

All data are representative at least three independent experiments. Data are expressed as mean ± standard deviation (SD). Statistical differences were analyzed using *t* test and one-way ANOVA with Bonferroni post hoc test using Prism 5 (GraphPad, San Diego, CA). A *P* value < 0.05 was considered statistically significant.

## Results

### *Slc7a11* and *Gpx4* expression was decreased in diabetic patient kidney tubules

**Fig. 1:**
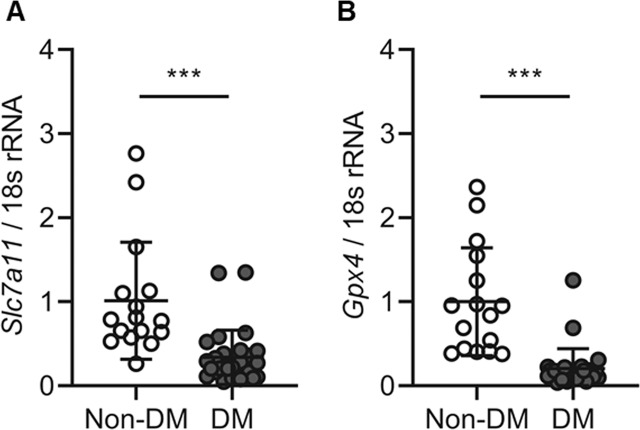
Expression of ferroptosis-related molecules *Slc7a11* and *Gpx4* was decreased in diabetic nephropathy of kidney biopsy tissues. **A**, **B** *Slc7a11* and *Gpx4* mRNA expression was significantly decreased in the DM group compared to the non-DM group. Bonferroni *t* tests were used for statistical analysis. Error bars represent SD. ***P < 0.001 versus non-DM group; non-DM group, *n* = 17; DM group, *n* = 28.

The baseline characteristics of the patients are shown in Table 1. The median age of the control and diabetic patients was 25 (18–55) and 52 (24–74) years, respectively. The mean DM duration of the patients was 10.96 (1–25) years, and 23 (82.1%) subjects had hypertension, whereas none tof the subjects without DM had HTN. When comparing subjects with and without DM, those with DM tended to have higher serum creatinine levels, higher serum BUN levels, and a higher UPCR. We assessed changes in ferroptosis-related molecules, such as xCT (*Slc7a11*) and GPX4 (*Gpx4*) in kidney biopsy samples from all study subjects. Kidney tubular *Slc7a11* and *Gpx4* mRNA expression was significantly lower in patients with DM than in non-DM patients (*P* < 0.001; Fig. 1A, B). These results suggest that ferroptosis is increased in kidney tubules of diabetic patients.

**Table 1:** Clinical parameters.

	Non-DM (*n* = 16)	DM (*n* = 28)
Sex, *n* (%)		
Men	14 (87.50)	17 (60.71)
Women	2 (12.50)	11 (39.29)
Age, years	25.00 ± 11.60	52.32 ± 12.05
BMI, kg/m^2^	22.03 ± 3.08	24.94 ± 4.28
HTN, *n* (%)	0.0 (0)	23 (82.14)
DM duration, years	—	10.96 ± 7.47
Serum creatinine, mg/dl	0.89 ± 0.12	2.62 ± 1.78
Serum BUN, mg/dl	13.09 ± 3.41	33.06 ± 14.05
eGFR, ml/min/1.73 m^2^	114.13 ± 18.19	40.71 ± 30.46
UPCR, g/g cr	0.29 ± 0.35	8.06 ± 5.68

Data are shown as mean ± standard deviation.

*BMI* body mass index, *HTN* hypertension, *DM* diabetes mellitus, *BUN* blood urea nitrogen, *eGFR* estimated glomerular filtration rate, *UPCR* urine protein to creatinine ratio.

### Kidney tubular cell death due to TGF-β1 is associated with upregulation of ferroptosis-related molecules

**Fig. 2:**
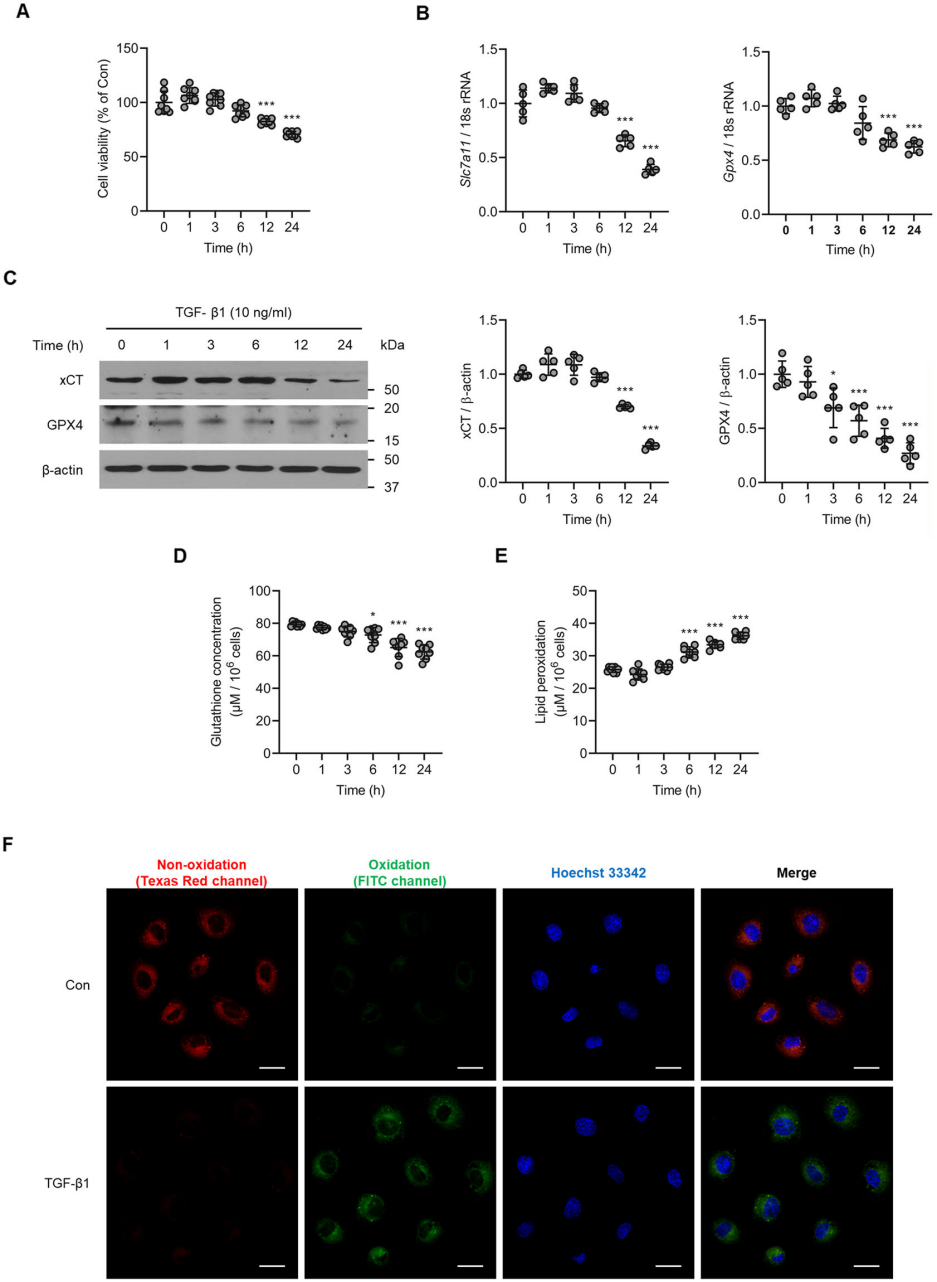
TGF-β1 induces kidney tubular cell death along with changes in ferroptosis-related molecules. **A** Cell viability measured via an MTT assay revealed a significant increase in the death of cultured NRK-52E cells exposed to TGF-β1 (10 ng/ml) in a time-dependent manner. **B** TGF-β1 caused a marked decrease in mRNA expression of ferroptosis-related molecules *Slc7a11* and *Gpx4* in NRK-52E cells. **C** Expression of xCT and GPX4 protein was significantly decreased in TGF-β1-stimulated NRK-52E cells compared to control cells. **D** Glutathione concentration was significantly decreased in cultured NRK-52E cells exposed TGF-β1 (10 ng/ml) after 6, 12, and 24 h. **E** TGF-β1 significantly induced lipid peroxidation in NRK-52E cells after 6, 12, and 24 h. **F** Lipid peroxidation assessed using image-iT^®^ revealed an increase in TGF-β1-treated NRK-52E cells after 12 h. As shown is representative of three independent replicates. One-way ANOVA and Bonferroni post hoc tests were used for statistical analysis. Error bars represent SD. Original magnification, ×40 for all. Scale bar = 20 µm. *P < 0.05; ***P < 0.001 versus 0 h group.

To explore cell death via ferroptosis in kidney cells, we analyzed NRK-52E cells with or without TGF-β1 (10 ng/ml) treatment. A cell viability assay revealed that cell death was significantly increased in TGF-β1-stimulated cells compared to control cells in a time-dependent manner (*P* < 0.05 for 12 h, *P* < 0.001 for 24 h; Fig. 2A). Next, we measured xCT and GPX4 expression, and found that TGF-β1 stimulation significantly reduced the expression of *Slc7a11* (*P* < 0.001, Fig. 2B) and *Gpx4* mRNA (*P* < 0.01 for 12 h, *P* < 0.001 for 24 h; Fig. 2B). Expression of xCT (*P* < 0.001 for 12 h, *P* < 0.01 for 24 h; Fig. 2C) and GPX4 (*P* < 0.001 for 6 h, *P* < 0.01 for 12 and 24 h; Fig. 2C) protein was also decreased in TGF-β1-stimulated cells. In addition, glutathione concentration was significantly decreased in NRK-52E cells exposed to TGF-β1 (*P* < 0.05 for 12 h, *P* < 0.01 for 24 h; Fig. 2D), whereas lipid peroxidation was significantly increased (*P* < 0.001 for 6, 12, and 24 h; Fig. 2E). Similar effects were observed with lipid peroxidation staining (Fig. 2F). Comparable findings were observed in Erastin-stimulated NRK-52E cells (Supplementary Fig. 1A–E). These results suggest that TGF-β1 reduces not only the influx of cysteine, which in turn diminishes glutathione synthesis, but also causes the degradation of lipid peroxide in TGF-β1-stimulated NRK-52E cells in a time-dependent manner. Both of these changes result in an accumulation of intracellular lipid peroxide, which ultimately leads to ferroptosis-induced cell death.

### Ferroptosis inhibitors abrogates TGF-β1-induced kidney tubular cell death

**Fig. 3:**
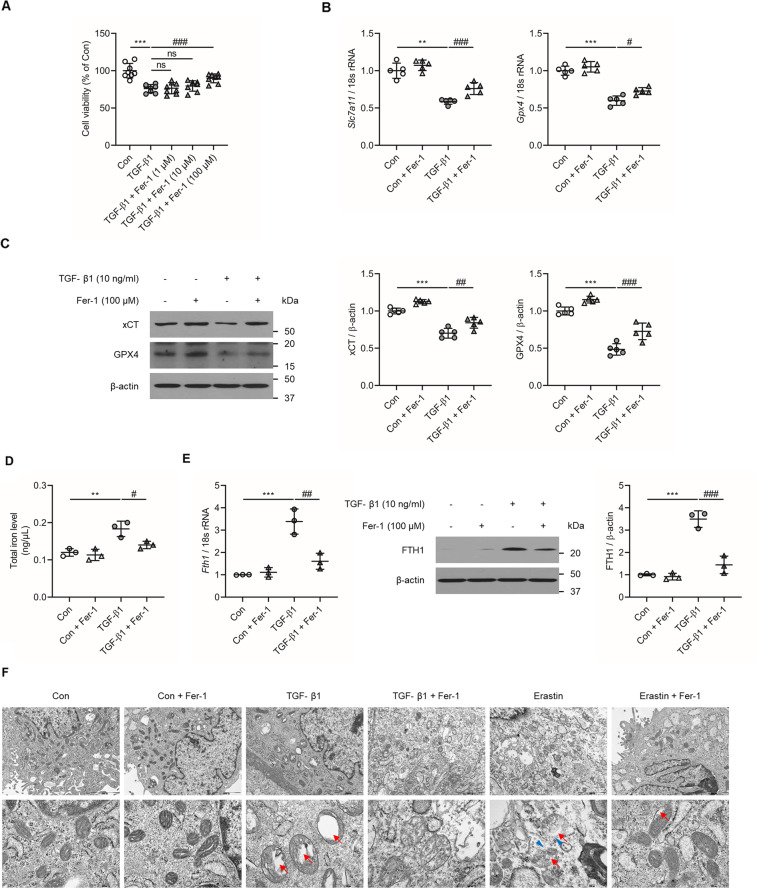
Effect of Fer-1 treatment on cell viability and changes in ferroptosis-related molecules in TGF-β1-stimulated NRK-52E cells at 12 h. **A** MTT assay revealed that administration of Fer-1 (100 µM) significantly abrogated the decrease in cell viability in cultured NRK-52E cells exposed to TGF-β1 (10 ng/ml). **B** The decrease in *Slc7a11* and *Gpx4* mRNA expression seen in TGF-β1-stimulated NRK-52E cells was significantly ameliorated after Fer-1 treatment. **C** The decrease in xCT and GPX4 protein expression seen in TGF-β1-stimulated NRK-52E cells was significantly attenuated after Fer-1 treatment. **D** The increase in total iron levels in TGF-β1-stimulated NRK-52E cells was significantly abrogated after Fer-1 treatment. **E** The increase in protein and mRNA expression levels of FTH1 in TGF-β1-stimulated NRK-52E cells was significantly attenuated after Fer-1 treatment. **F** Transmission electron microscope images of mitochondria. Red arrow indicates mitochondria cristae vanish. Blue arrowhead indicates outer mitochondria membrane rupture. As shown is representative of three independent replicates. One-way ANOVA and Bonferroni post hoc tests were used for statistical analysis. Error bars represent SD. Original magnification, ×150 for upper and ×600 for lower. Scale bar = 2000 µm (upper), 500 µm (lower). **P < 0.01; ***P < 0.001 versus Con group. ^#^*P* < 0.05; ^##^*P* < 0.01; ^###^*P* < 0.001 versus TGF-β1 group.

Next, we investigated the impact of Fer-1, an inhibitor of the ferroptosis pathway, on TGF-β1-induced kidney tubular cell death. Administration of Fer-1 (100 µM) significantly ameliorated the decrease in the viability of TGF-β1-stimulated NRK-52E cells (*P* < 0.001; Fig. 3A). Expression of *Slc7a11* (*P* < 0.001, Fig. 3B) and *Gpx4* (*P* < 0.05, Fig. 3B) mRNA was significantly attenuated by Fer-1 in TGF-β1-stimulated NRK-52E cells. In addition, the expression of xCT (*P* < 0.01, Fig. 3C) and GPX4 (*P* < 0.001, Fig. 3C) protein was also significantly abrogated by Fer-1 administration NRK-52E cells. Furthermore, total iron levels and mRNA and protein levels of FTH1 was significantly increased in TGF-β1-treated NRK-52E cells compared to controls (Figs. 3D, E). The increase in total iron level, and FTH1 expression level was abrogated with Fer-1 treatment. A similar finding was noted in NRK-52E cells stimulated with the ferroptosis inducing positive control Erastin (Supplementary Fig. 2A–E). When the mitochondria morphologic features were observed through TEM, TGF-β1-treated NRK-52E cells revealed features of mitochondria shrinkage and vanishing of mitochondrial cristae. These findings were comparable to the Erastin-induced positive control ferroptosis morphologic features. These noted changes of the mitochondria were attenuated with the Fer-1 treatment (Fig. 3F).

**Fig. 4:**
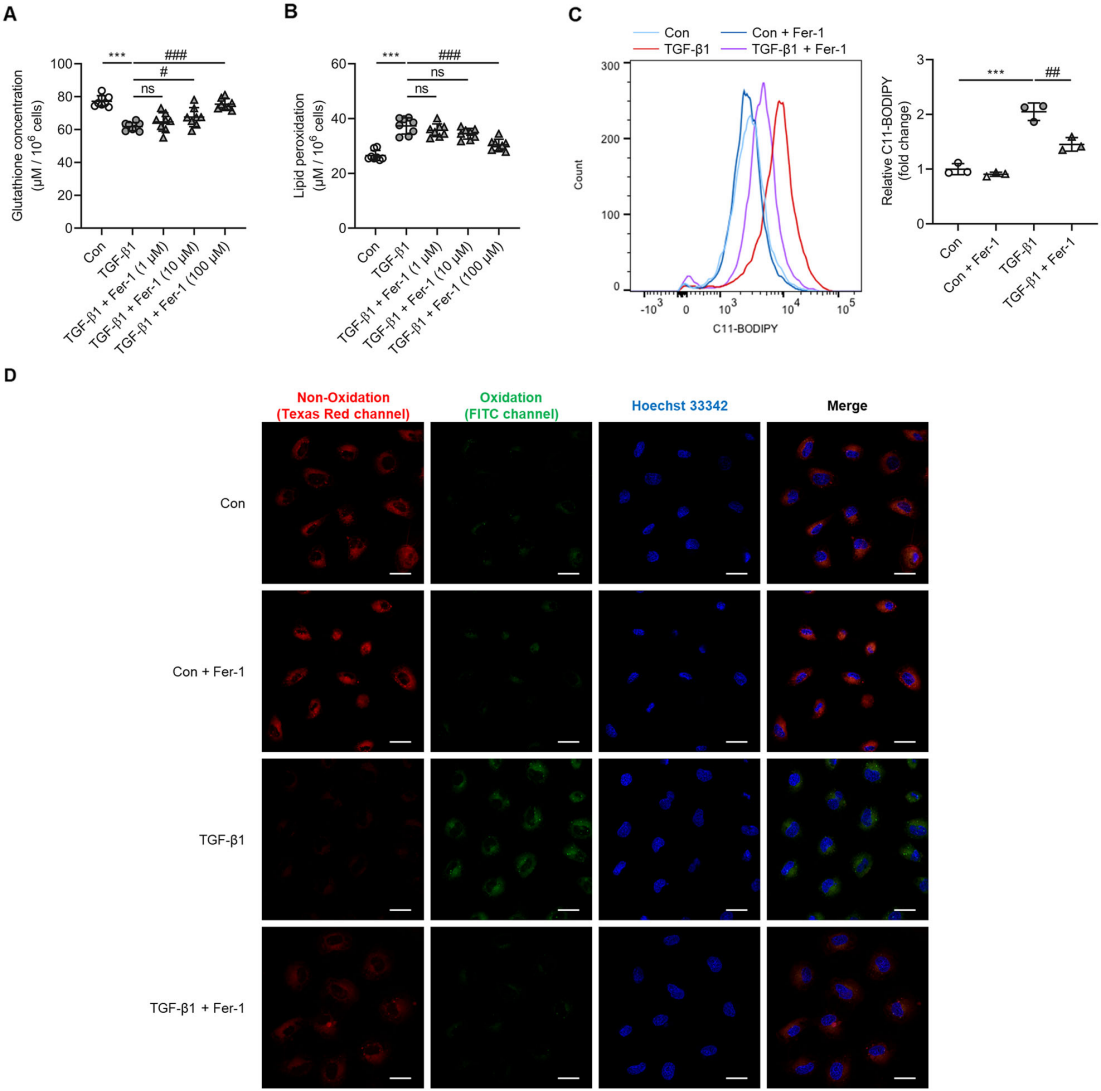
Ferroptosis inhibitors attenuated glutathione concentration and lipid peroxidation in TGF-β1-stimulated NRK-52E cells after 12 h. **A** The decrease in glutathione concentration seen in cultured NRK-52E cells exposed TGF-β1 (10 ng/ml) was significantly ameliorated by Fer-1 (10 and 100 µM) treatment. **B** Administration of Fer-1 significantly attenuated the increase in lipid peroxidation seen in TGF-β1-stimulated NRK-52E cells. **C** FACS evaluated BODIPY 581/591-C11 fluorescence intensity. **D** Lipid peroxidation assessed using Image-iT^®^ revealed that the increase seen in cultured NRK-52E cells after exposure to TGF-β1 was significantly abrogated by Fer-1 treatment. As shown is representative of three independent replicates. One-way ANOVA and Bonferroni post hoc tests were used for statistical analysis. Error bars represent SD. Original magnification, ×40 for all. Scale bar = 20 µm. ***P < 0.001 versus Con group. ^#^*P* < 0.05; ^##^*P* < 0.01; ^###^*P* < 0.001 versus TGF-β1 group.

The decrease in glutathione concentration caused by TGF-β1 was ameliorated by Fer-1 treatment (*P* < 0.05 versus Fer-1 10 µM, *P* < 0.001 versuss Fer-1 100 µM; Fig. 4A). Lipid peroxidation was attenuated by administration of Fer-1 100 µM (*P* < 0.001; Figs. 4B–D). In addition, Fer-1 abrogated lipid peroxidation in Erastin-stimulated NRK-52E cells (Supplementary Fig. 3A–C). Taken together, these findings suggest that ferroptosis is involved in TGF-β1-induced tubular cell death.

### Fer-1 protects against kidney injury in an experimental model of diabetes

In the animal experiments, body weight was significantly lower in the STZ-induced diabetic group (22.33 ± 1.73 g) than the Con group (30.32 ± 3.61 g; *P* < 0.001; Table 2), and this was improved following treatment with Fer-1 (26.81 ± 2.71 g; *P* < 0.001; Table 2), at the end of the 12-week study period. In contrast, kidney weight and the ratio of kidney weight to body weight were significantly higher in the STZ group than the Con group (*P* < 0.05 for kidney weight, *P* < 0.001 for kidney/body weight; Table 2), and again, these effects were rescued by administration of Fer-1 (*P* < 0.05 for kidney weight, *P* < 0.001 for kidney/body weight; Table 2). Mean blood glucose levels were significantly higher in the STZ (524.00 ± 84.18 mg/dl) and STZ + Fer-1 (515.70 ± 94.51 mg/dl) groups than the Con group (126.20 ± 17.50 mg/dl; *P* < 0.001; Table 2). Moreover, the ratio of urinary albumin to creatinine was significantly higher in the STZ group (0.053 ± 0.017 µg/mg) than the Con group (0.015 ± 0.004 µg/mg; *P* < 0.001; Table 2), and this was attenuated by Fer-1 treatment (0.033 ± 0.010 µg/mg; *P* < 0.05; Table 2). The elevated serum BUN and creatinine levels in the STZ group, compared to the Con group, showed tendencies of attenuation in the STZ + Fer-1 group (Table 2).

**Table 2:** Kidney function parameters in animal model.

	Con	Con + Fer-1	STZ	STZ + Fer-1
Body weight, g	30.32 ± 3.61	29.33 ± 1.54	22.33 ± 1.73	26.81 ± 2.71
Kidney weight, mg	188.40 ± 12.23	177.55 ± 7.65	197.05 ± 14.41	186.10 ± 11.49
Kidney/body weight, 10^−3^	6.28 ± 0.62	6.07 ± 0.43	8.84 ± 0.40	6.98 ± 0.46
Blood glucose, mg/dl	126.20 ± 17.50	114.70 ± 18.87	524.00 ± 84.18	515.70 ± 94.51
Serum creatinine, mg/dl	0.15 ± 0.04	0.13 ± 0.02	0.24 ± 0.09	0.20 ± 0.04
Serum BUN, mg/dl	24.92 ± 2.42	23.00 ± 1.17	29.26 ± 2.80	26.90 ± 4.12
UACR, µg/mg	0.015 ± 0.004	0.013 ± 0.001	0.053 ± 0.017	0.033 ± 0.010

Data are shown as mean ± standard deviation.

*Con* control, *Fer-1* ferrostatin-1, *DM* diabetes mellitus, *BUN* blood urea nitrogen, *UACR* urine albumin to creatinine ratio.

**Fig. 5:**
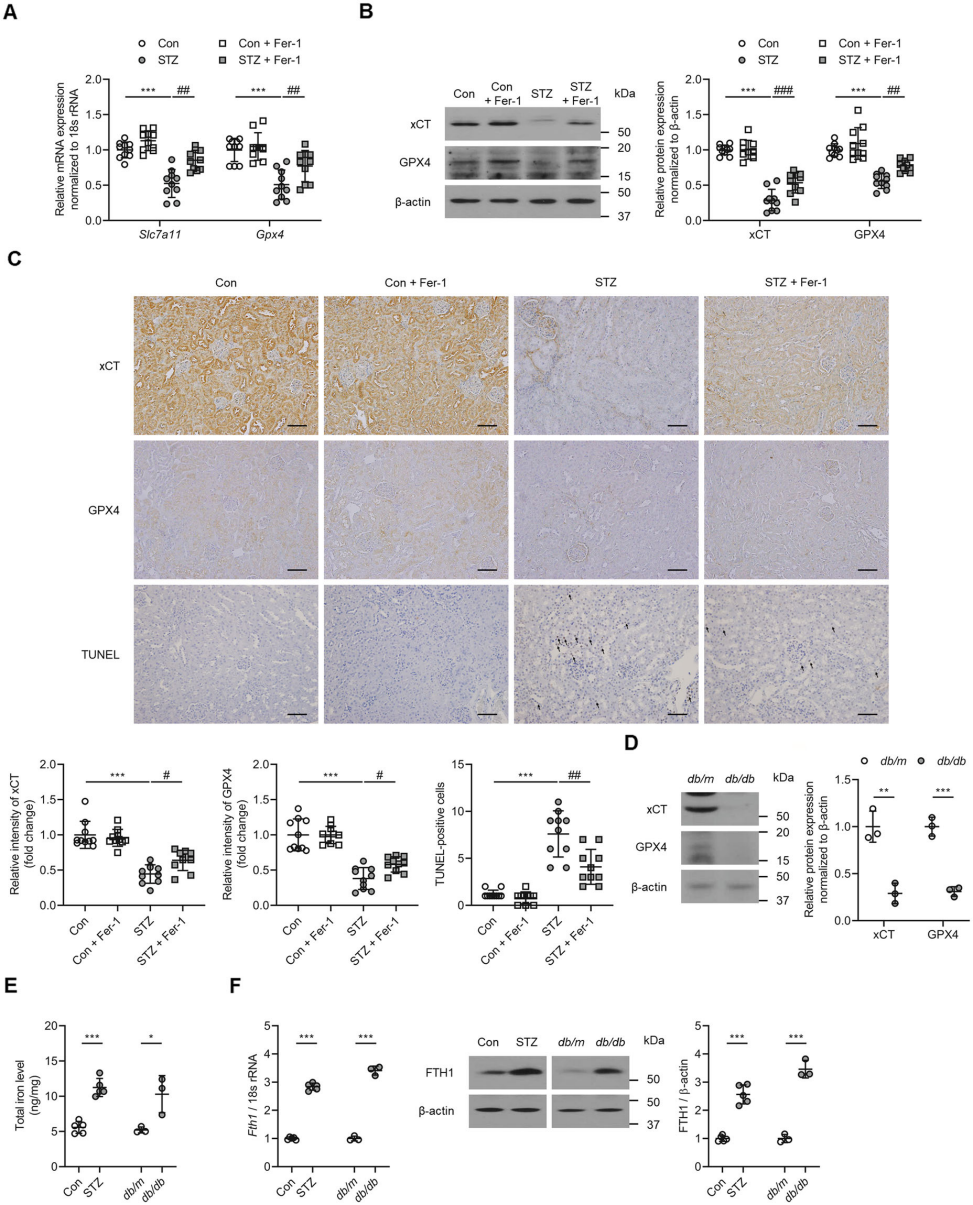
Effect of Fer-1 on cell viability and changes in ferroptosis-related molecules in the kidney of diabetic mice. **A** Kidney *Slc7a11* and *Gpx4* mRNA expression was significantly lower in STZ mice than the Con group, and changes in the STZ kidney were abrogated by Fer-1 treatment. **B** xCT and GPX4 protein expression assessed by western blot was significantly lower in the STZ kidney, and this was ameliorated by administration of Fer-1. **C** TUNEL staining showed a significant increase in cell death in the STZ kidney, which was alleviated by Fer-1 treatment. **D** Protein and mRNA expression of xCT and GPX4 in kidney samples of *db/db* mice. **E** Total iron levels measured in kidney samples of STZ and *db/db* mice. **F** Protein and mRNA expression level of FTH1 in kidney samples of STZ and *db/db* mice. One-way ANOVA and Bonferroni post hoc tests were used for statistical analysis. Error bars represent SD. Original magnification, ×20 for all. Scale bar = 100 µm. **P < 0.01; ***P < 0.001 versus Con group. ^#^*P* < 0.05; ^##^*P* < 0.01; ^###^*P* < 0.001 versus STZ group. *n* = 10 for each group of STZ. *n* = 3 for each group of *db/db*.

Next, we sought to determine whether ferroptosis was induced in the kidneys of the experimental diabetic animals and whether Fer-1 could mitigate the kidney injury induced by DM. The expression of xCT and GPX4 was significantly lower (*P* < 0.001 for *Slc7a11*, xCT, and GPX4; *P* < 0.01 for *Gpx4*; Fig. 5A–C), while cell death, assessed by a TUNEL assay, was significantly higher in the kidneys of STZ mice (*P* < 0.001; Fig. 5C) than in the kidneys of Con mice. The effects on xCT (*Slc7a11*) and GPX4 (*Gpx4*) expression, as well as cell death were all significantly improved following Fer-1-treatment (*P* < 0.05; Fig. 5A–C). In addition, total iron level and mRNA and protein levels of FTH1 was significantly increased in the kidneys of STZ and *db/db* mice compared to control and *db/m* mice, respectively (Figs. 5D–F).

**Fig. 6:**
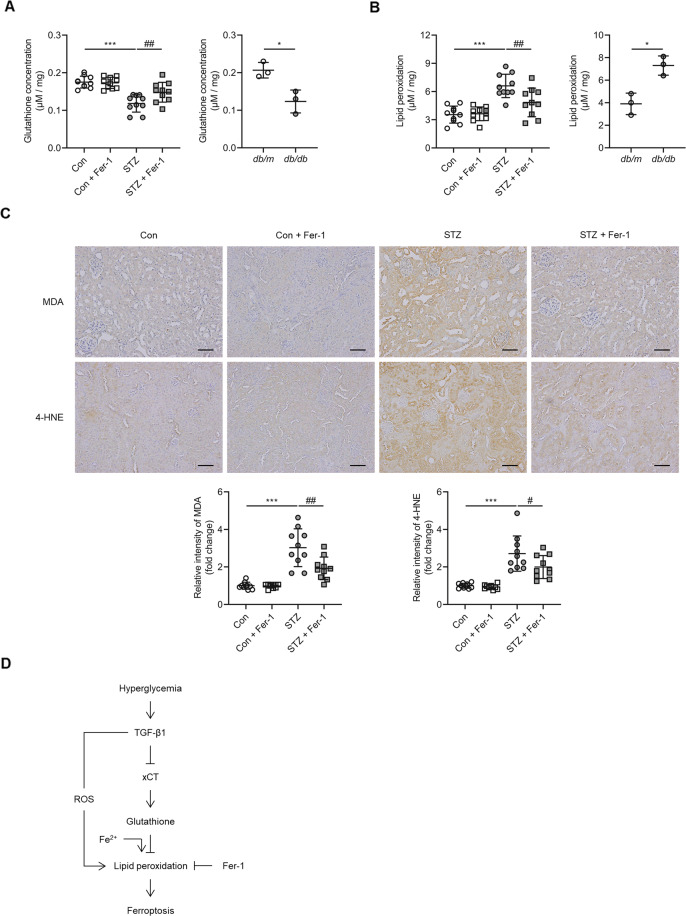
Effect of Fer-1 on glutathione concentration and lipid peroxidation in the kidney of diabetic mice. **A** Glutathione concentration in the kidneys of STZ and *db/db* mice. **B** Lipid peroxidation assessed by MDA levels in the kidneys of STZ and *db/db* mice. **C** Immunohistochemistry for MDA and 4-HNE was significantly increased in the STZ group, and this was abrogated by administration of Fer-1. **D** Schematic diagram of TGF-β1-induced ferroptosis in diabetic nephropathy. One-way ANOVA and Bonferroni post hoc tests were used for statistical analysis. Error bars represent SD. Original magnification, ×20 for all. Scale bar = 100 µm. *P < 0.05; ***P < 0.001 versus Con group. ^#^*P* < 0.05; ^##^*P* < 0.01 versus STZ group; ROS reactive oxygen species. *n* = 10 for each group of STZ. *n* = 3 for each group of *db/db*.

Kidney glutathione concentration was significantly decreased (*P* < 0.001; Fig. 6A), whereas lipid peroxidation was significantly increased (*P* < 0.001; Fig. 6B) in the kidneys of animals in the STZ group, and these changes were ameliorated following administration of Fer-1 (*P* < 0.05 for glutathione concentration, *P* < 0.01 for lipid peroxidation; Fig. 6A, B). Also, MDA and 4-HNE, lipid peroxidation markers, were significantly attenuated after Fer-1-treatment (*P* < 0.001 for MDA, *P* < 0.05 for 4-HNE; Fig. 6C). The decrease in glutathione concentration and increase in lipid peroxidation was also observed in the kidneys of *db/db* when compared to *db/m* mice (Figs. 6A, B). Together, these findings demonstrate that ferroptosis is induced in experimental diabetic nephropathy through the effect of TGF-β1 and that treatment with Fer-1, a ferroptosis inhibitor, attenuated kidney cell death in diabetic mice (Fig. 6D).

### Tubular expression of NRF2 and p53 in diabetic nephropathy

In order to assess the relationship between ferroptosis and NRF2 and p53, the expression levels of NRF2 and p53 were further evaluated. The p53 and nuclear NRF2 protein and levels were significantly increased in TGF-β1-stimulated NRK-52E cells than control cells (Supplementary Fig. 4A). These findings were comparable to the Erastin-induced positive control ferroptosis features (Supplementary Fig. 4B). A similar increase in p53 and nuclear NRF2 protein levels were also noted in the kidneys of STZ and *db/db* mice compared to control and *db/m* mice (Supplementary Fig. 4C, D).

## Discussion

In the present study, we found that *Slc7a11* and *Gpx4* mRNA expression was lower in DM patients than non-DM patients. We also demonstrated that xCT and GPX4 expression was significantly lower in cultured tubular epithelial cells that were exposed to TGF-β1, as well as in the kidneys of diabetic mice, resulting in low glutathione levels and enhanced lipid peroxidation. Moreover, administration of the ferroptosis inhibitor, Fer-1, significantly abrogated not only the changes in glutathione levels and lipid peroxidation, but also cell death in TGF-β1-stimulated cultured tubular cells. Fer-1 also significantly ameliorated kidney hypertrophy and albuminuria, and reduced intrarenal accumulation of lipid peroxidation in the diabetic mice.

In diabetic nephropathy, kidney dysfunction accompanied by nephron loss has been attributed to inflammation, ECM accumulation, and tubular cell death, which is also seen in nephrotoxic and ischemia-reperfusion injury models^27,28^. Meanwhile, since the proximal tubule requires high energy and relies on aerobic metabolism, a consequence of increased consumption, impaired utilization, and reduced delivery of O_2_, makes it vulnerable to ischemic injury in diabetes^29^. Therefore, tubular cell death has been recognized as one of the key findings in kidney damage of various etiologies. In particular, in addition to apoptosis, recent reports have shown regulated necrosis to play a role in the pathogenesis of AKI^30^. Based on these findings, the possibility of regulated necrosis, including necroptosis, pyroptosis, and ferroptosis, to contribute in diabetes-associated tubular cell has been proposed. However, most of the previous studies on tubular cell death under diabetic conditions have only addressed apoptotic cell death^14,15,31^.

Ferroptosis is a form of regulated cell death characterized by the iron-dependent accumulation of lipid hydroperoxides such that they reach lethal levels. It was first described by Dixon et al. as a type of cell death induced by the small-molecule Erastin, which inhibits xCT^16^. xCT imports cystine into cells, where it is further reduced to cysteine^32^. Cysteine in subsequently converted to glutathione by the addition of glutamate and glycine^33^. Glutathione is essential for the function of GPX4, which converts potentially toxic lipid hydroperoxides to nontoxic lipid alcohols and as a result, prevents the accumulation of lipid reactive oxygen species^34^. Therefore, a decrease in xCT inhibits the intracellular import of cysteine, leading to less glutathione synthesis, lower GPX4 activity, and ultimately the accumulation of lipid hydroperoxides, which is linked to loss of plasma membrane integrity^35^. To date, only a limited number of studies have investigated changes in xCT and GPX4 expression under diabetic conditions. Carpi-Santos et al. found that xCT subunit protein expression is significantly decreased in high glucose-stimulated cultured retinal cells and retinas of STZ-induced diabetic rats, and this is accompanied by lower glutathione levels and an increase in oxidative stress^36^. Furthermore, a recent study showed that patients with diabetes exhibit significantly lower levels of GPX4 enzyme in their heart, than age-matched nondiabetic patients^37^. In accordance with results from previous studies, we demonstrated that xCT and GPX4 mRNA and protein expression, both of which are key molecules involved in ferroptosis, were significantly decreased in cultured tubular epithelial cells exposed to TGF-β1 and in the kidneys of STZ mice, suggesting that these changes may contribute to the decrease in glutathione concentration and the increase in lipid peroxidation.

The manifestations of diabetic nephropathy are known to be a consequence of the actions of certain cytokines and growth factors. Of these, TGF-β1 is considered as the key player of diabetic nephropathy development in both type 1 and type 2 DM^38^. The high glucose stimulation in diabetic conditions increase TGF-β1 expression in kidney cells, which promotes cellular hypertrophy and matrix production^39^. Accordingly, TGF-β1 stimulation has been widely used as an effective diabetic nephropathy in vitro model. The results found with TGF-β1 stimulation in this study were comparable to the features found in kidney tubular cells treated with the positive control ferroptosis inducer Erastin, suggesting that ferroptosis may be induced in kidney tubular cells under diabetic conditions. These results are in line with a recent investigation which has shown that TGF-β1 represses xCT expression via Smad3 activation, and enhances lipid peroxidation in hepatocellular carcinoma cells^40^. Since TGF-β1 is known to increase reactive oxygen species production^41^, the accumulation of oxidative stress through TGF-β1 stimulation may lead to ferroptosis development. In addition, there could also be a possibility in which TGF-β1 directly inhibits the cystine/glutamate antiporter xCT. However, further evaluations would be needed to elucidate the precise mechanism.

To date, a number of studies have suggested that ferroptosis plays a pathological role in the development of degenerative brain disorders, including Parkinson’s^42^, Huntington’s^43,44^, and Alzheimer’s disease^45,46^, as well as in diverse types of cancer^47,48^ and AKI^21–23^. Furthermore, ferroptosis inhibitors such as Fer-1, which is a novel potent first generation small molecule, inhibit ferroptosis by blocking lipid peroxidation and thus ameliorate pathologic abnormalities in various diseases. In addition, a novel third-generation ferrostatin (termed 16–86) was recently generated and was shown to be more stable and potent than the first-in-class compound Fer-1 (ref. ^22^). In the present study, when Fer-1 was used to inhibit ferroptosis TGF-β1-induced tubular cell death was attenuated and lipid peroxidation was reduced, suggesting that ferroptosis may play a role in the development of diabetic tubulopathy. Cell death within kidney tissue was assessed via TUNEL staining in the current study, which has been widely used to examine in vivo cell death in various tissues^49–52^. Since double-strand breaks are found in most cells undergoing programmed cell death, the majority of cells will be positive for TUNEL staining^53^. However, a TUNEL-positive signal is not limited to apoptotic cells, and cells that have undergone regulated necrosis will also be TUNEL positive^54^. One way to differentiate apoptosis from regulated necrosis is to perform immunohistochemical staining for cleaved caspase-3 in addition to TUNEL staining; however, this was not done in the present study.

Tubular atrophy and tubulointerstitial fibrosis are the main pathological features associated with kidney dysfunction in patients with diabetic nephropathy^55^. Compared to glomerular or vascular lesions, tubulointerstitial lesions were found to be more useful for predicting renal prognosis in DM patients^56–58^. Even though the underlying pathways that mediate the development of tubulointerstitial lesions in diabetic nephropathy are not well understood, previous studies have suggested that TGF-β1 is the principal mediator of the deleterious effects of the diabetic milieu^11^. Furthermore, the impact of TGF-β1 on oxidative stress, ECM synthesis, and cell viability is significantly more powerful than that of high glucose^7,59,60^. Thus, in the current study, we used normal glucose medium plus TGF-β1 rather than high glucose medium for the cell culture experiments.

In summary, the results of present study provide evidence that glutathione levels are significantly decreased in TGF-β1-stimulated kidney tubular cells and in the kidneys of diabetic mice, and this is accompanied by a significant reduction in xCT and GPX4 expression and a significant increase in lipid peroxidation. Furthermore, these changes are associated with cell death under diabetic conditions, which is significantly ameliorated by ferroptosis inhibition. These findings suggest that ferroptosis plays a significant role in the development of diabetic nephropathy.

## Supplementary information

Supplementary figure legends

Supplementary figure 1

Supplementary figure 2

Supplementary figure 3

Supplementary figure 4

## References

[1] Brenner BM (2001). Effects of losartan on renal and cardiovascular outcomes in patients with type 2 diabetes and nephropathy. N. Engl. J. Med..

[2] McClellan WM (2011). Albuminuria and racial disparities in the risk for ESRD. J. Am. Soc. Nephrol..

[3] D D’Agati V, Fogo AB, Bruijn JA, Jennette JC (2004). Pathologic classification of focal segmental glomerulosclerosis: a working proposal. Am. J. Kidney Dis..

[4] Mason RM, Wahab NA (2003). Extracellular matrix metabolism in diabetic nephropathy. J. Am. Soc. Nephrol..

[5] Morais C, Westhuyzen J, Pat B, Gobe G, Healy H (2005). High ambient glucose is effect neutral on cell death and proliferation in human proximal tubular epithelial cells. Am. J. Physiol. Ren. Physiol..

[6] Ginevri F (1993). Reversible tubular proteinuria precedes microalbuminuria and correlates with the metabolic status in diabetic children. Pediatr. Nephrol..

[7] Dronavalli S, Duka I, Bakris GL (2008). The pathogenesis of diabetic nephropathy. Nat. Rev. Endocrinol..

[8] Sanchez-Niño M-D, Benito-Martin A, Ortiz A (2010). New paradigms in cell death in human diabetic nephropathy. Kidney Int..

[9] Elmarakby AA, Sullivan JC (2012). Relationship between oxidative stress and inflammatory cytokines in diabetic nephropathy. Cardiovasc. Ther..

[10] Guha M, Xu ZG, Tung D, Lanting L, Natarajan R (2007). Specific down-regulation of connective tissue growth factor attenuates progression of nephropathy in mouse models of type 1 and type 2 diabetes. FASEB J..

[11] Böttinger EP (2002). & Bitzer, M. TGF-ß signaling in renal disease. J. Am. Soc. Nephrol..

[12] Dai C, Yang J, Liu Y (2003). Transforming growth factor-β1 potentiates renal tubular epithelial cell death by a mechanism independent of Smad signaling. J. Biol. Chem..

[13] Kato M, Natarajan R (2014). Diabetic nephropathy–emerging epigenetic mechanisms. Nat. Rev. Nephrol..

[14] Kumar D, Robertson S, Burns KD (2004). Evidence of apoptosis in human diabetic kidney. Mol. Cell Biochem..

[15] Shahzad K (2016). Caspase-1, but not caspase-3, promotes diabetic nephropathy. J. Am. Soc. Nephrol..

[16] Dixon SJ (2012). Ferroptosis: an iron-dependent form of nonapoptotic cell death. Cell.

[17] Dixon SJ, Stockwell BR (2014). The role of iron and reactive oxygen species in cell death. Nat. Chem. Biol..

[18] Yang WS, Stockwell BR (2016). Ferroptosis: death by lipid peroxidation. Trends Cell Biol..

[19] Gaschler MM, Stockwell BR (2017). Lipid peroxidation in cell death. Biochem. Biophys. Res. Commun..

[20] Louandre C (2013). Iron‐dependent cell death of hepatocellular carcinoma cells exposed to sorafenib. Int. J. Cancer.

[21] Martin-Sanchez D (2017). Ferroptosis, but not necroptosis, is important in nephrotoxic folic acid–induced AKI. J. Am. Soc. Nephrol..

[22] Linkermann A (2014). Synchronized renal tubular cell death involves ferroptosis. Proc. Natl Acad. Sci. USA.

[23] Angeli JPF (2014). Inactivation of the ferroptosis regulator Gpx4 triggers acute renal failure in mice. Nat. Cell Biol..

[24] Tang S (2002). Free-hand, ultrasound-guided percutaneous renal biopsy: experience from a single operator. Eur. J. Radio..

[25] Adler SG (2001). Glomerular mRNAs in human type 1 diabetes: biochemical evidence for microalbuminuria as a manifestation of diabetic nephropathy. Kidney Int..

[26] Kang SW, Adler SG, Lapage J, Natarajan R (2001). p38 MAPK and MAPK kinase 3/6 mRNA and activities are increased in early diabetic glomeruli. Kidney Int..

[27] Mohan S (2013). Recovery of renal function among ESRD patients in the US Medicare program. PLoS ONE.

[28] Yokoyama H (2000). Higher incidence of diabetic nephropathy in type 2 than in type 1 diabetes in early-onset diabetes in Japan. Kidney Int..

[29] Gilbert RE (2017). Proximal tubulopathy: prime mover and key therapeutic target in diabetic kidney disease. Diabetes.

[30] Basile DP, Anderson MD, Sutton TA (2012). Pathophysiology of acute kidney injury. Compr. Physiol..

[31] Kato M (2009). TGF-beta activates Akt kinase through a microRNA-dependent amplifying circuit targeting PTEN. Nat. Cell Biol..

[32] Mandal PK (2010). System xc-and thioredoxin reductase 1 cooperatively rescue glutathione deficiency. J. Biol. Chem..

[33] Lu SC (2013). Glutathione synthesis. Biochim. Biophys. Acta.

[34] Feng H, Stockwell BR (2018). Unsolved mysteries: how does lipid peroxidation cause ferroptosis?. PLoS Biol..

[35] Cao JY, Dixon SJ (2016). Mechanisms of ferroptosis. Cell Mol. Life Sci..

[36] Carpi-Santos R (2016). Early changes in system xc− and glutathione in the retina of diabetic rats. Exp. Eye Res..

[37] Katunga LA (2015). Obesity in a model of gpx4 haploinsufficiency uncovers a causal role for lipid-derived aldehydes in human metabolic disease and cardiomyopathy. Mol. Metab..

[38] Ziyadeh FN (1998). Evidence for the involvement of transforming growth factor-β in the pathogenesis of diabetic kidney disease: Are Koch’s postulates fulfilled?. Curr. Pr. Med..

[39] Li JH, Huang XR, Zhu HJ, Johnson R, Lan HY (2003). Role of TGF-beta signaling in extracellular matrix production under high glucose conditions. Kidney Int..

[40] Kim DH, Kim WD, Kim SK, Moon DH, Lee SJ (2020). TGF-β1-mediated repression of SLC7A11 drives vulnerability to GPX4 inhibition in hepatocellular carcinoma cells. Cell Death Dis..

[41] Rhyu DY (2005). Role of reactive oxygen species in TGF-beta1-induced mitogen-activated protein kinase activation and epithelial-mesenchymal transition in renal tubular epithelial cells. J. Am. Soc. Nephrol..

[42] Guiney SJ, Adlard PA, Bush AI, Finkelstein DI, Ayton S (2017). Ferroptosis and cell death mechanisms in Parkinson’s disease. Neurochem. Int..

[43] Sadagurski M (2011). IRS2 increases mitochondrial dysfunction and oxidative stress in a mouse model of Huntington disease. J. Clin. Investig..

[44] Chen J (2013). Iron accumulates in Huntington’s disease neurons: protection by deferoxamine. PLoS ONE.

[45] Di Domenico F, Tramutola A, Butterfield DA (2017). Role of 4-hydroxy-2-nonenal (HNE) in the pathogenesis of alzheimer disease and other selected age-related neurodegenerative disorders. Free Radic. Biol. Med..

[46] Chiang GC (2017). Relationships among cortical glutathione levels, brain amyloidosis, and memory in healthy older adults investigated in vivo with 1H-MRS and Pittsburgh compound-B PET. ANJR Am. J. Neuroradiol..

[47] Yang WS (2014). Regulation of ferroptotic cancer cell death by GPX4. Cell.

[48] Eling N, Reuter L, Hazin J, Hamacher-Brady A, Brady NR (2015). Identification of artesunate as a specific activator of ferroptosis in pancreatic cancer cells. Oncoscience.

[49] Gobe G (2009). Identification of apoptosis in kidney tissue sections. Methods Mol. Biol..

[50] Healy E, Dempsey M, Lally C, Ryan MP (1998). Apoptosis and necrosis: mechanisms of cell death induced by cyclosporine A in a renal proximal tubular cell line. Kidney Int..

[51] Gavrieli Y, Sherman Y, Ben-Sasson SA (1992). Identification of programmed cell death in situ via specific labeling of nuclear DNA fragmentation. J. Cell Biol..

[52] Phillips LM (2011). The renin inhibitor aliskiren attenuates high-glucose induced extracellular matrix synthesis and prevents apoptosis in cultured podocytes. Nephron. Exp. Nephrol..

[53] Elmore S (2007). Apoptosis: a review of programmed cell death. Toxicol. Pathol..

[54] de Torres C, Munell F, Ferrer I, Reventós J, Macaya A (1997). Identification of necrotic cell death by the TUNEL assay in the hypoxic-ischemic neonatal rat brain. Neurosci. Lett..

[55] Gilbert RE, Cooper ME (1999). The tubulointerstitium in progressive diabetic kidney disease: more than an aftermath of glomerular injury?. Kidney Int..

[56] Tang SC, Leung JC, Lai KN (2011). Diabetic tubulopathy: an emerging entity. Contrib. Nephrol..

[57] Yu Y (2010). Urinary biomarkers trefoil factor 3 and albumin enable early detection of kidney tubular injury. Nat. Biotechnol..

[58] Bonventre JV (2012). Can we target tubular damage to prevent renal function decline in diabetes. Semin. Nephrol..

[59] Iglesias-de la Cruz MC (2001). Hydrogen peroxide increases extracellular matrix mRNA through TGF-β in human mesangial cells. Kidney Int..

[60] Kanwar YS (2008). Diabetic nephropathy: mechanisms of renal disease progression. Exp. Biol. Med..

