# Correction: Characterization of ferroptosis in kidney tubular cell death under diabetic conditions

**DOI:** 10.1038/s41419-021-03667-y

**Published:** 2021-04-08

**Authors:** Seonghun Kim, Shin-Wook Kang, Jeongho Joo, Seung Hyeok Han, Huiyoon Shin, Bo Young Nam, Jimin Park, Tae-Hyun Yoo, Gyuri Kim, Pureunchowon Lee, Jung Tak Park

**Affiliations:** 1 Department of Oral Pathology, Oral Cancer Research Institute, College of Dentistry, Yonsei University, Seoul, South Korea; 2 Institute of Kidney Disease Research, College of Medicine, Yonsei University, Seoul, South Korea; 3 Department of Internal Medicine, College of Medicine, Yonsei University, Seoul, South Korea; 4 Genome & Health Big Data Laboratory, Seoul National University, Seoul, South Korea; 5 Severance Biomedical Science Institute, College of Medicine, Yonsei University, Seoul, South Korea

**Keywords:** Cell death, Kidney diseases

Correction to: *Cell Death and Disease*

10.1038/s41419-021-03452-x, published online 08 February 2021

The original version of this article unfortunately contained a mistake. After the publication of the article the authors noticed that the order of Supplementary information figures was mixed up in the online published version. The original article has been corrected.

## Supplementary Information

Supplementary figure legends

Supplementary figure 1

Supplementary figure 2

Supplementary figure 3

Supplementary figure 4

