# Supplementary material for: Characterization of ferroptosis in kidney tubular cell death under diabetic conditions
Source: Cell Death Dis. 2021 Feb 8;12(2):160. doi: 10.1038/s41419-021-03452-x (PMC7870666; doi:10.1038/s41419-021-03452-x)
Supplement: Supplementary file 1 — Supplementary figure legends [file 41419_2021_3452_MOESM1_ESM.docx]

**Supplementary Figure Legends**

**Figure S1. Erastin induced ferroptosis in NRK-52E cells.** (A) Cell viability measured via an MTT assay revealed a significant increase in the death of cultured NRK-52E cells exposed to Erastin in a dose-dependent manner. (B) Glutathione concentration was significantly decreased in cultured NRK-52E cells exposed Erastin after 12 h. (C) Erastin significantly induced lipid peroxidation in NRK-52E cells after 12 h. (D) Erastin caused a marked decrease in mRNA expression of ferroptosis-related molecules *Slc7a11* and *Gpx4* in NRK-52E cells. (E) Expression of xCT and GPX4 protein was significantly decreased in Erastin-stimulated NRK-52E cells compared to control cells. As shown is representative of three independent replicates. One-way ANOVA and Bonferroni *post hoc* tests were used for statistical analysis. Error bars represent SD. ** P < 0.01; *** P < 0.001 versus Erastin 0 µM group.

**Figure S2. Effect of Fer-1 in Erastin-stimulated NRK-52E cells at 12 h.** (A) MTT assay revealed that administration of Fer-1 significantly abrogated the decrease in cell viability in cultured NRK-52E cells exposed to Erastin dose-dependent manner. (B) The decrease in *Slc7a11* and *Gpx4* mRNA expression seen in Erastin-stimulated NRK-52E cells was significantly ameliorated after Fer-1 treatment. (C) The decrease in xCT and GPX4 protein expression seen in Erastin-stimulated NRK-52E cells was significantly attenuated after Fer-1 treatment. (D) The increase in total iron levels in Erastin-stimulated NRK-52E cells was significantly abrogated after Fer-1 treatment. (E) The increase in Protein and mRNA expression levels of FTH1 in Erastin-stimulated NRK-52E cells was significantly attenuated after Fer-1 treatment. As shown is representative of three independent replicates. One-way ANOVA and Bonferroni *post hoc* tests were used for statistical analysis. Error bars represent SD. ** P < 0.01; *** P < 0.001 versus Con group. # P < 0.05; ### P < 0.001 versus Erastin group.

**Figure S3. Fer-1 attenuated lipid peroxidation in Erastin-stimulated NRK-52E cells at 12 h.** (A) Administration of Fer-1 significantly attenuated the increase in lipid peroxidation seen in Erastin-stimulated NRK-52E cells. (B) FACS evaluated BODIPY 581/591-C11 fluorescence intensity. (C) Lipid peroxidation assessed using Image-iT^®^ revealed that the increase seen in cultured NRK-52E cells after exposure to Erastin was significantly abrogated by Fer-1 treatment. As shown is representative of three independent replicates. One-way ANOVA and Bonferroni *post hoc* tests were used for statistical analysis. Error bars represent SD. Original magnification, x40 for all. Scale bar = 20 µm. *** P < 0.001 versus Con group. ###P < 0.001 versus Erastin group.

**Figure S4. p53 and nuclear NRF2 protein expression in TGF-β1 or Erastin-stimulated NRK-52E cells and kidney tissue.** (A and B) p53 and nuclear NRF2 protein expression was significantly increased in TGF-β1 or Erastin-stimulated NRF-52E cells. (C and D) p53 and nuclear NRF2 protein expression was significantly increased in STZ or *db/db* mice. As shown of *in vitro* study is representative of three independent replicates. Bonferroni *t* tests were used for statistical analysis. Error bars represent SD. * P < 0.05; ** P < 0.01; *** P < 0.001 versus Con group; STZ group, n = 5; *db/db* group, n = 3.
