# Supplementary figures and images for: Characterization of ferroptosis in kidney tubular cell death under diabetic conditions
Source: Cell Death Dis. 2021 Feb 8;12(2):160. doi: 10.1038/s41419-021-03452-x (PMC7870666; doi:10.1038/s41419-021-03452-x)

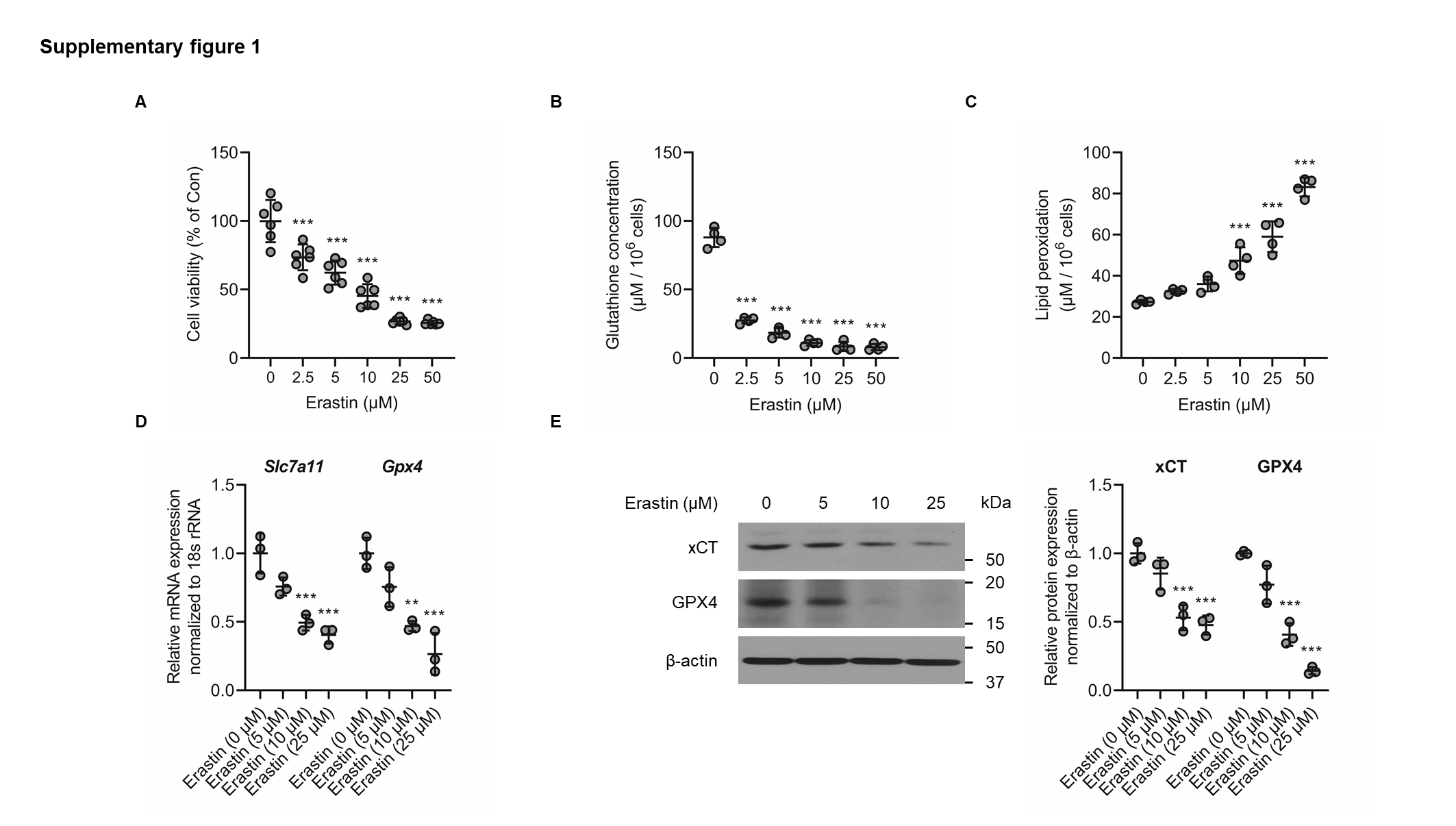

Supplement: Supplementary file 2 — Supplementary figure 1 [file 41419_2021_3452_MOESM2_ESM.tif]

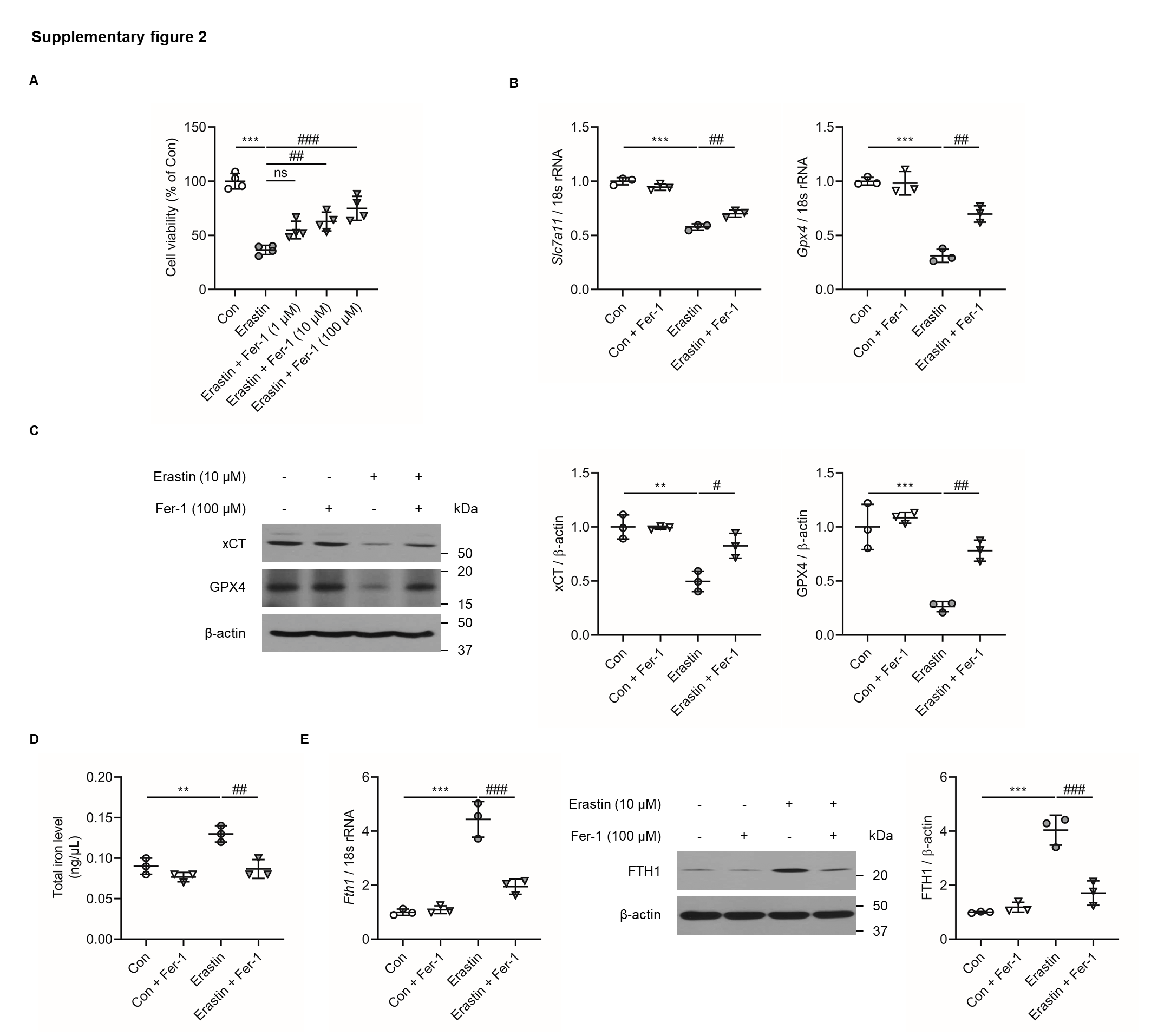

Supplement: Supplementary file 3 — Supplementary figure 2 [file 41419_2021_3452_MOESM3_ESM.tif]

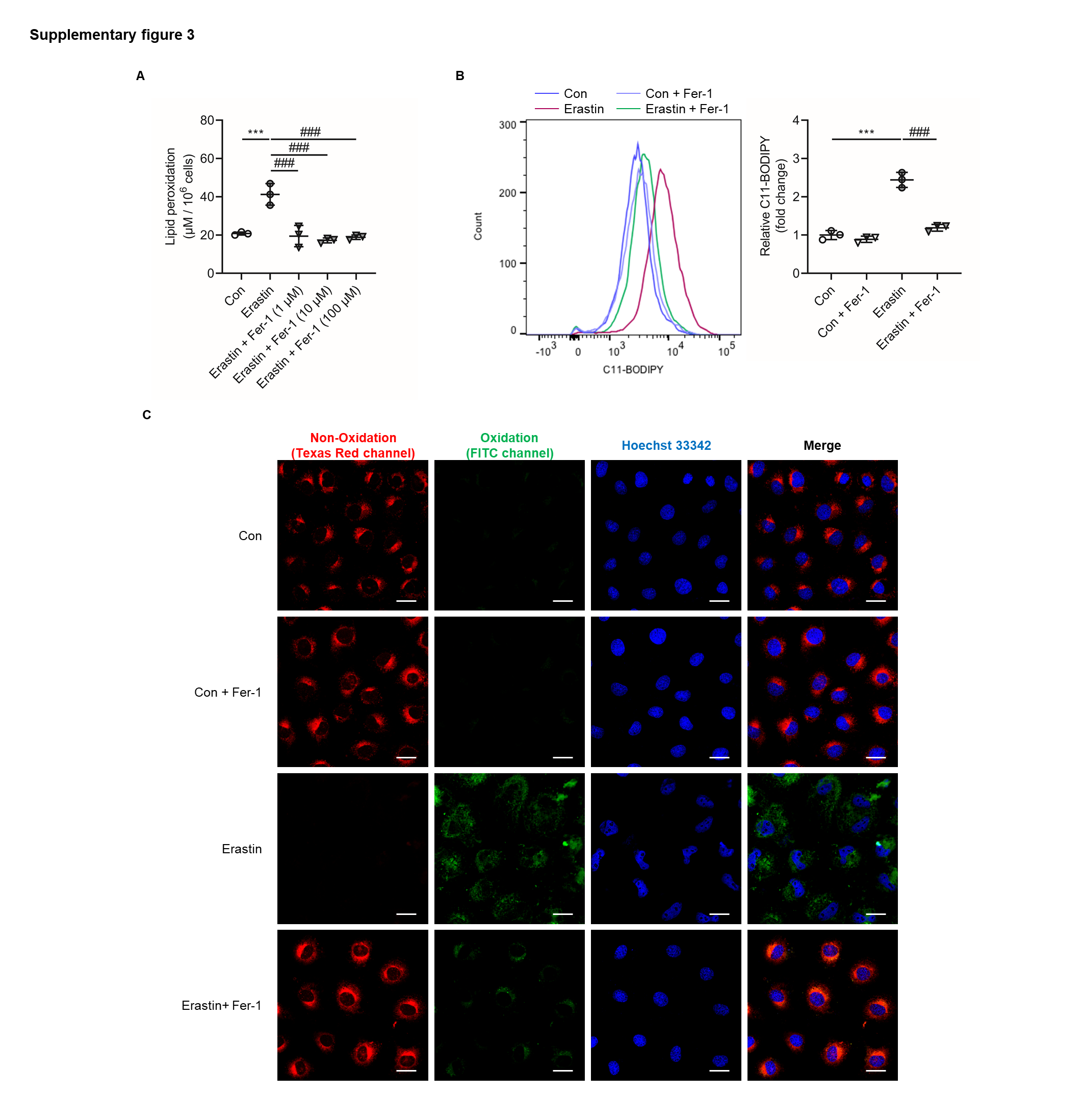

Supplement: Supplementary file 4 — Supplementary figure 3 [file 41419_2021_3452_MOESM4_ESM.tif]

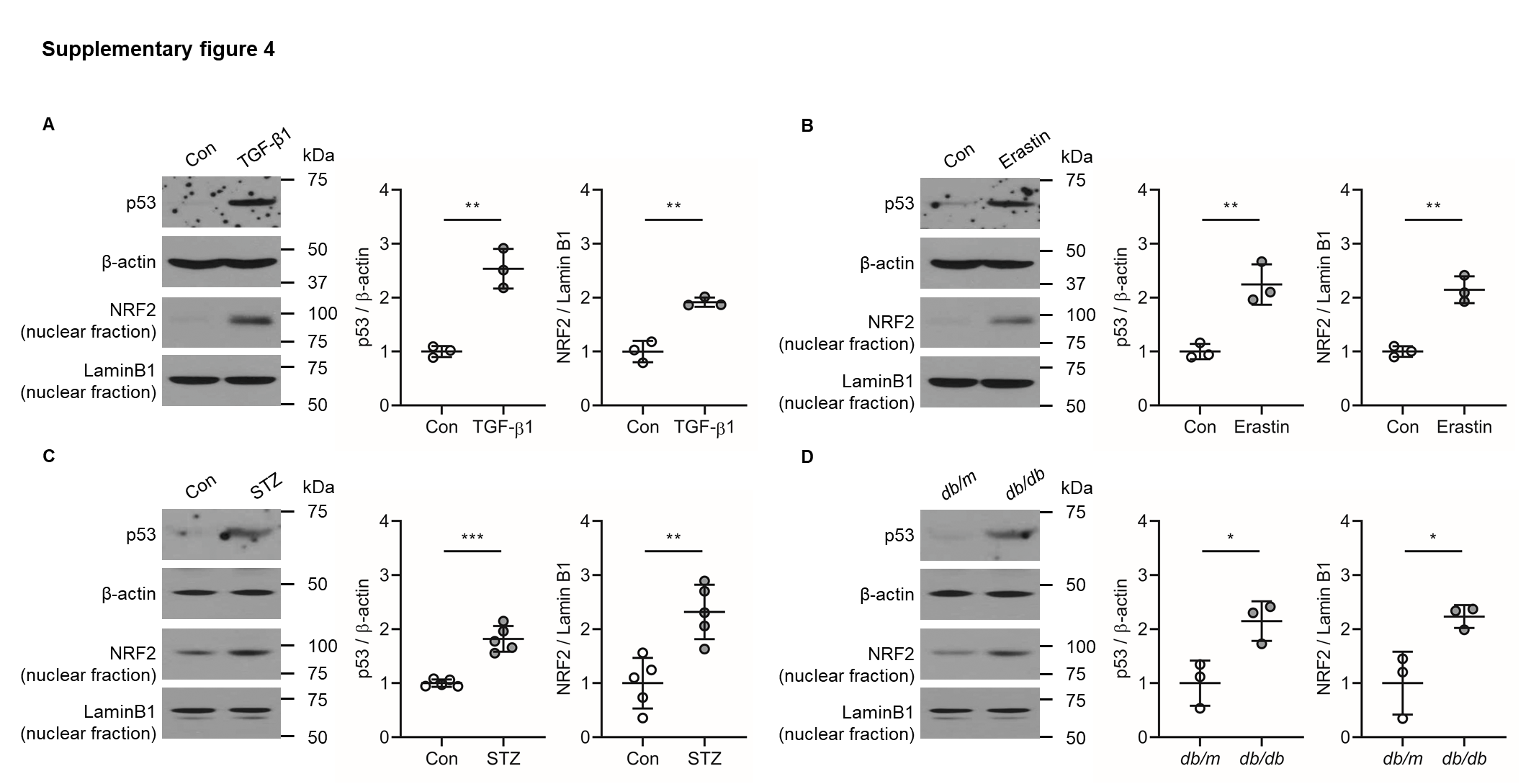

Supplement: Supplementary file 5 — Supplementary figure 4 [file 41419_2021_3452_MOESM5_ESM.tif]
